# The Salisbury Treatment of Consumption

**Published:** 1879-12

**Authors:** 


### EDITORIAL, ETC.

*The Salisbury Treatment of Consumption.*—We make no apology for the publication of this paper. With over one-fourth of our adult population dying of phthisis, it is an object to increase the general interest felt in any method which may lead to better results in the treatment of this fearful and yet so common malady. So many dentists become consumptive that we are sure the readers of this *Journal* will be glad to know of a method which, by food only, is proving itself a success.

To test the question thoroughly, Dr. E. Cutter, of Boston, whose original papers of late have aided in filling these columns, himself a steadfast believer in the food treatment, has established a "sanatarium" near Boston, and solicits the aid of physicians in the procuring of cases. Those who send him chronic cases of consumption will be constituted a committee to whom he will report results in six months, *themselves to be the judges of the success or failure* of the treatment. Dr. Cutter is in this acting disinterestedly, and wishes to test this matter thoroughly; with of course, all confidence in its success.

If any reader of this *Journal* suffering from pulmonary disease, desires the comforts of a home, and treatment on principles seemingly thoroughly rational, let him write to Dr. C. at 94 Tremont Street, Boston. This notice is entirely unsolicited on his part, and written as a felt duty to the many hopelessly perishing. H.

